# Striatal Dopaminergic Depletion Pattern Reflects Pathological Brain Perfusion Changes in Lewy Body Diseases

**DOI:** 10.1007/s11307-022-01745-x

**Published:** 2022-06-14

**Authors:** Yu Iwabuchi, Tohru Shiga, Masashi Kameyama, Raita Miyazawa, Morinobu Seki, Daisuke Ito, Hiroyuki Uchida, Hajime Tabuchi, Masahiro Jinzaki

**Affiliations:** 1 Department of Radiology, Keio University School of Medicine, Tokyo, Japan; 2 Advanced Clinical Research Center, Fukushima Global Medical Science Center, Fukushima Medical University, Fukushima, Japan; 3 Department of Diagnostic Radiology, Tokyo Metropolitan Geriatric Hospital and Institute of Gerontology, Tokyo, Japan; 4 Department of Neurology, Keio University School of Medicine, Tokyo, Japan; 5 Department of Physiology, Keio University School of Medicine, Tokyo, Japan; 6 Department of Neuropsychiatry, Keio University School of Medicine, Tokyo, Japan

**Keywords:** α-synuclein, α-synucleinopathy, ^123^I-FP-CIT, SPM, Parkinsonian syndrome, Parkinsonism

## Abstract

**Purpose:**

In Lewy body diseases (LBD), various symptoms occur depending on the distribution of Lewy body in the brain, and the findings of brain perfusion and dopamine transporter single-photon emission computed tomography (DAT-SPECT) also change accordingly. We aimed to evaluate the correlation between brain perfusion SPECT and quantitative indices calculated from DAT-SPECT in patients with LBD.

**Procedures:**

We retrospectively enrolled 35 patients with LBD who underwent brain perfusion SPECT with N-isopropyl-p-[^123^I] iodoamphetamine and DAT-SPECT with ^123^I-ioflupane. Mini-mental state examination (MMSE) data were also collected from 19 patients. Quantitative indices (specific binding ratio [SBR], putamen-to-caudate ratio [PCR], and caudate-to-putamen ratio [CPR]) were calculated using DAT-SPECT. These data were analysed by the statistical parametric mapping procedure.

**Results:**

In patients with LBD, decreased PCR index correlated with hypoperfusion in the brainstem (medulla oblongata and midbrain) (uncorrected *p* < 0.001, *k* > 100), while decreased CPR index correlated with hypoperfusion in the right temporoparietal cortex (family-wise error corrected *p* < 0.05), right precuneus (uncorrected *p* < 0.001, *k* > 100), and bilateral temporal cortex (uncorrected *p* < 0.001, *k* > 100). However, there was no significant correlation between decreased SBR index and brain perfusion. Additionally, the MMSE score was correlated with hypoperfusion in the left temporoparietal cortex (uncorrected *p* < 0.001).

**Conclusions:**

This study suggests that regional changes in striatal ^123^I-ioflupane accumulation on DAT-SPECT are related to brain perfusion changes in patients with LBD.

## Introduction

Lewy body diseases (LBD), which are sequential neurodegenerative disorders and have many common clinical features and neuropathology, are considered subtypes of an α-synuclein-associated disease spectrum from incidental LBD and non-demented Parkinson’s disease (PD) to PD with dementia (PDD) and dementia with Lewy bodies (DLB). Although the nosologic relationship among these disorders is frequently being debated, it is currently difficult to classify them explicitly. Based on the conventional international consensus, DLB is diagnosed when cognitive impairment precedes parkinsonian motor symptoms or begins 1 year after the onset of parkinsonian; in contrast, in PDD, cognitive impairment develops in the setting of well-established PD [1]. Therefore, the diagnosis of LBD depends on the presence and timing of onset of cognitive impairment. Braak et al. proposed that LB pathology spreads via the olfactory bulb or gastrointestinal system from the peripheral to central nervous systems, and LB travels from the brainstem to the cortex [2]. The distribution and degree of LB pathology in the brain affect clinical symptoms in patients with LBD, including movement disorder, psychotic state, visual hallucination, and cognitive impairment, in patients with LBD.

Nuclear medicine imaging is useful in diagnosing LBD. Brain perfusion single-photon emission computed tomography (SPECT) shows changes in brain perfusion in this patient group. Notably, patients with PDD and DLB show parietal, temporal, or occipital hypoperfusion, while those with PD show no remarkable deterioration in brain perfusion compared with those with PDD or DLB [3]. These changes are related to cognitive impairment in these patients [4]. In addition, dopamine transporter (DAT)-SPECT is another useful nuclear medicine imaging approach to assess the function of the presynaptic nigrostriatal dopaminergic system. Quantitative indices calculated from DAT-SPECT are also useful in interpreting and assessing images [5, 6]. In patients with LBD, striatal tracer accumulation deteriorates as the disease condition progresses [7, 8]. The specific binding ratio (SBR), representing the strength of striatal tracer accumulation, is the most frequently used quantitative index in DAT-SPECT. Although the SBR index is useful because it correlates with the severity of motor dysfunction in patients with PD [9], it is not enough to differentiate between patients with PD and DLB [10]. On the other hand, the putamen-to-caudate (PCR) and caudate-to-putamen (CPR) ratios are different types of quantitative indices of DAT-SPECT that represent changes in the shape and distribution of striatal tracer accumulation. Accumulating studies have demonstrated that the PCR/CPR indices can differentiate patients with PD and DLB [10–12]. It is speculated that the anterior part of striatal uptake, especially caudate uptake, correlates with psychotic state or cognitive impairment, while the posterior part of striatal uptake correlates with movement disorders in patients with LBD.

We hypothesised that changes in striatal tracer accumulation on DAT-SPECT would correlate with brain perfusion in patients with LBD and that these changes would reflect their symptoms, such as movement disorder or cognitive impairment, caused by differential distributions of LB pathology in the brain. Accordingly, this study aimed to assess the correlation between brain perfusion and quantitative indices (SBR, PCR, and CPR indices) of DAT-SPECT using the statistical parametric mapping (SPM) analysis and to compare these quantitative indices among patients with LBD, including PD, PDD, and DLB.

## Materials and Methods

### Patients

A total of 231 consecutive patients who underwent brain perfusion SPECT with N-isopropyl-p-[^123^I] iodoamphetamine (IMP) and DAT-SPECT with ^123^I-ioflupane from February 2014 to January 2019 were included in this retrospective study. The interval between the two examinations was less than 1 year. Patients with a disease that affects the image quality of DAT and brain perfusion SPECT (e.g., extensive cerebral haemorrhage and infarction) were excluded. Enrolled patients were diagnosed based on the clinical diagnostic criteria of the UK Parkinson’s Disease Society Brain Bank [13] or established diagnostic criteria [1, 14]. Patients who were clinically undiagnosed with LBD were also excluded. General cognitive function was assessed in some of the participants using the mini-mental state examination (MMSE). Of the total 231 patients, 135 overlapped with those in our previous studies [10, 15].

The institutional review board of Keio University School of Medicine granted permission for this retrospective review of clinical data and imaging and waived the requirement for obtaining informed consent from the patients (approval number: 20211068).

### Image Acquisition and Reconstruction

Fifteen minutes after injection of 222 MBq of ^123^I-IMP, brain perfusion SPECT were obtained on Discovery NM 630 or Discovery NM/CT 670 (GE Healthcare, Milwaukee, WI) equipped with an extended low-energy general-purpose collimator. Projection data were acquired for 30 min. Imaging parameters were as follows: matrix size, 128 × 128; pixel size, 2.9 mm; slice thickness, 2.9 mm; and energy window, 159 keV ± 10%. Data were reconstructed by the filtered back-projection method with a Butterworth filter (critical frequency, 0.45; power, 10.0). Attenuation correction was used, while scatter correction was not.

Three hours after injection of 185 MBq ^123^I-ioflupane, DAT-SPECT were obtained on the Discovery NM 630 or Discovery NM/CT 670 (GE Healthcare, Milwaukee, WI) equipped with a FAN beam collimator. Projection data were acquired for 30 min. Imaging parameters were as follows: matrix size, 128 × 128; pixel size, 4.4 mm; slice thickness, 4.4 mm; and energy window, 159 keV ± 10%. Data were reconstructed by the ordered-subset expectation–maximisation method (iterations, 3; subset, 10) with a Butterworth filter (critical frequency, 0.5; power, 10.0). Neither attenuation correction nor scatter correction was used.

### Image Analysis of DAT-SPECT

We used commercially available software—DaTQUANT (GE Healthcare, Little Chalfont, UK) for calculation of quantitative indices of DAT-SPECT. DaTQUANT applies a normalised volume of interest (VOI) template, two striatal VOIs and two occipital lobe VOIs, based on the large European multi-centre database of healthy controls (ENC-DAT trial) (Fig. 2) [16, 17]. DaTQUANT enables setting these normalised VOIs automatically and calculation of the different types of quantitative indices: the SBR and PCR [10, 15, 18]. The SBR is defined as the mean counts of the striatal VOI (background-subtracted) divided by the mean counts of the occipital lobe VOI. The PCR is defined as the mean counts of the putamen VOI divided by the mean counts of the caudate VOI. We also calculated the CPR index, which is defined as the mean counts of the caudate VOI divided by the mean counts of the putamen VOI, as the simple reciprocal of the PCR index. In this study, the average values of the SBR and PCR/CPR for both sides of the striatum were used for the analysis.

### Statistical Model

The Kruskal–Wallis test was used to compare age, quantitative indices (SBR, PCR, and CPR), and the MMSE between the LBD (PD, PDD, and DLB) groups. If there were significant differences, post hoc analysis with Bonferroni correction was performed. The Pearson’s chi-square test was used to compare sex between these groups. These tests were performed using SPSS software (version 27; SPSS Inc., Chicago, IL).

Brain perfusion imaging data were preprocessed and analysed with statistical parametric mapping (SPM) software (https://www.fil.ion.ucl.ac.uk/spm/software/spm12/), running on the Matlab R2020a software environment (MathWorks, Natick, MA). Brain perfusion SPECT data were first aligned and anatomically standardised to match each scan to the Montreal Neurological Institute (MNI) atlas based template (McGill University, Montreal, Canada) using a 12-parameter affine transformation, followed by non-linear transformations [19, 20]. SPECT images were then smoothed with an 8 × 8 × 8 mm Gaussian filter. Normalisation of global brain counts to a value of 50 was performed with proportional scaling to remove differences in global activity in and between subjects. Proportional scaling was performed by dividing each voxel value by the average value of the whole parenchyma [21, 22]. Linear regression analyses after adjusting for age were conducted to determine the correlations between brain perfusion and quantitative indices calculated from DAT-SPECT and between brain perfusion and MMSE scores.

The initial voxel threshold was set to 0.001 uncorrected for multiple comparisons. Clusters were considered significant when falling below a cluster-corrected *p* (family-wise error [FWE]) = 0.05. If statistical significance was not reached, the threshold at the voxel level was explored at *p* < 0.001 uncorrected.

## Results

**Fig. 1:**
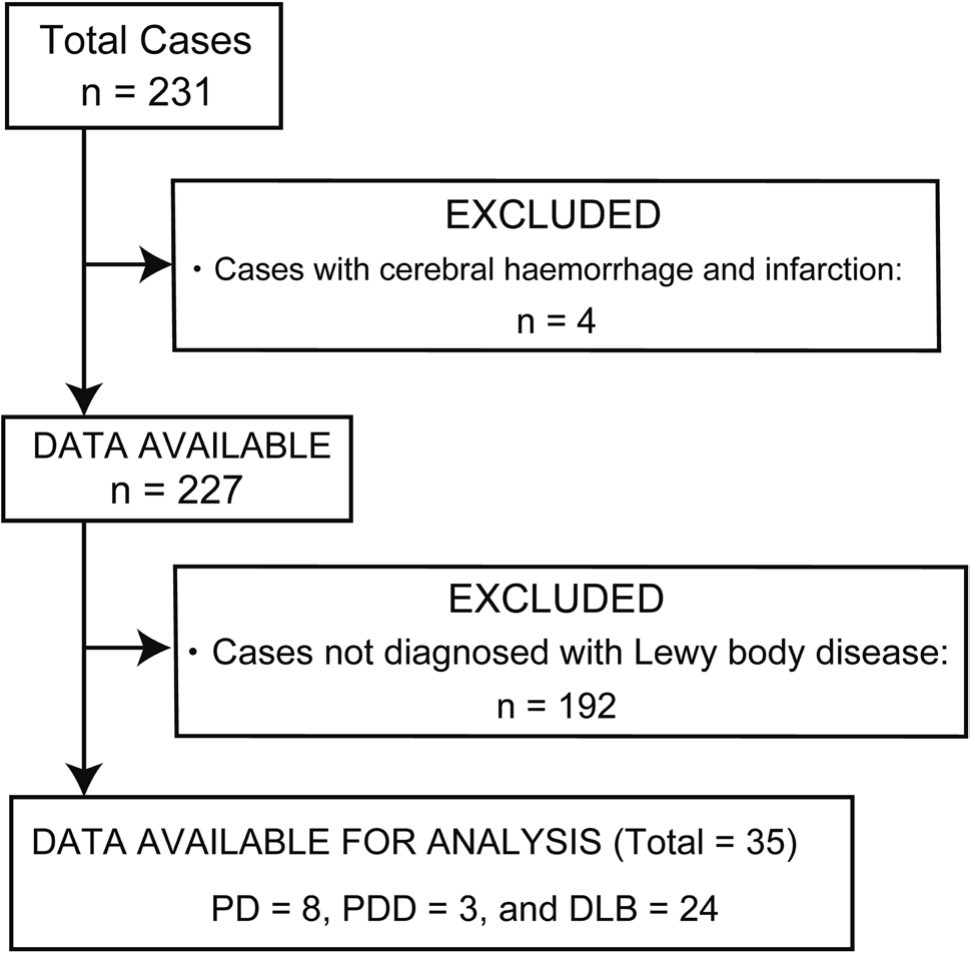
Flow diagram of patient inclusion. DLB, dementia with Lewy body; PD, Parkinson’s disease; PDD, Parkinson’s disease with dementia

Of the included 231 consecutive patients, four were excluded from the study due to insufficient image quality because of extensive cerebral haemorrhage and infarction, along with 192 who were clinically undiagnosed with LBD. The remaining 35 patients (median age, 76.0 years; range, 58–91 years; men/women, 13/22), including eight with PD, three with PDD, and 24 with DLB, were included in this analysis. Figure 1 depicts the flow diagram of study participants.

Table 1 shows the characteristics of included patients. The means and standard deviations of the SBR, PCR, and CPR indices and MMSE scores are also shown. Significant differences were observed in age, and the PCR and CPR indices between the PD and DLB groups.

**Table 1:** Patient characteristics

Characteristics	PD	PDD	DLB
n	8	3	24
Age (years, mean ± SD)	67.1 ± 5.9*	80.3 ± 1.5	78.5 ± 6.6*
Sex (male/female)	2/6	1/2	10/14
Quantitative indices from DAT-SPECT			
SBR	1.18 ± 0.50	1.07 ± 0.86	1.09 ± 0.44
PCR	0.73 ± 0.08*	0.89 ± 0.16	0.89 ± 0.12*
CPR	1.38 ± 0.14*	1.15 ± 0.22	1.15 ± 0.16*
MMSE (*n*)	27.0 ± 2.8 (2)	24.0 ± 1.0 (3)	21.8 ± 5.5 (14)

*CPR*, caudate-to-putamen ratio; *DAT*, dopamine transporter; *DLB*, dementia with Lewy body; *MMSE*, mini-mental state examination; *PCR*, putamen-to-caudate ratio; *PD*, Parkinson’s disease; *PDD*, Parkinson’s disease with dementia; *SBR*, specific binding ratio; *SD*, standard deviation; *SPECT*, single-photon emission computed tomography

^*^ *p* < 0.05 PD vs. DLB

**Fig. 2:**
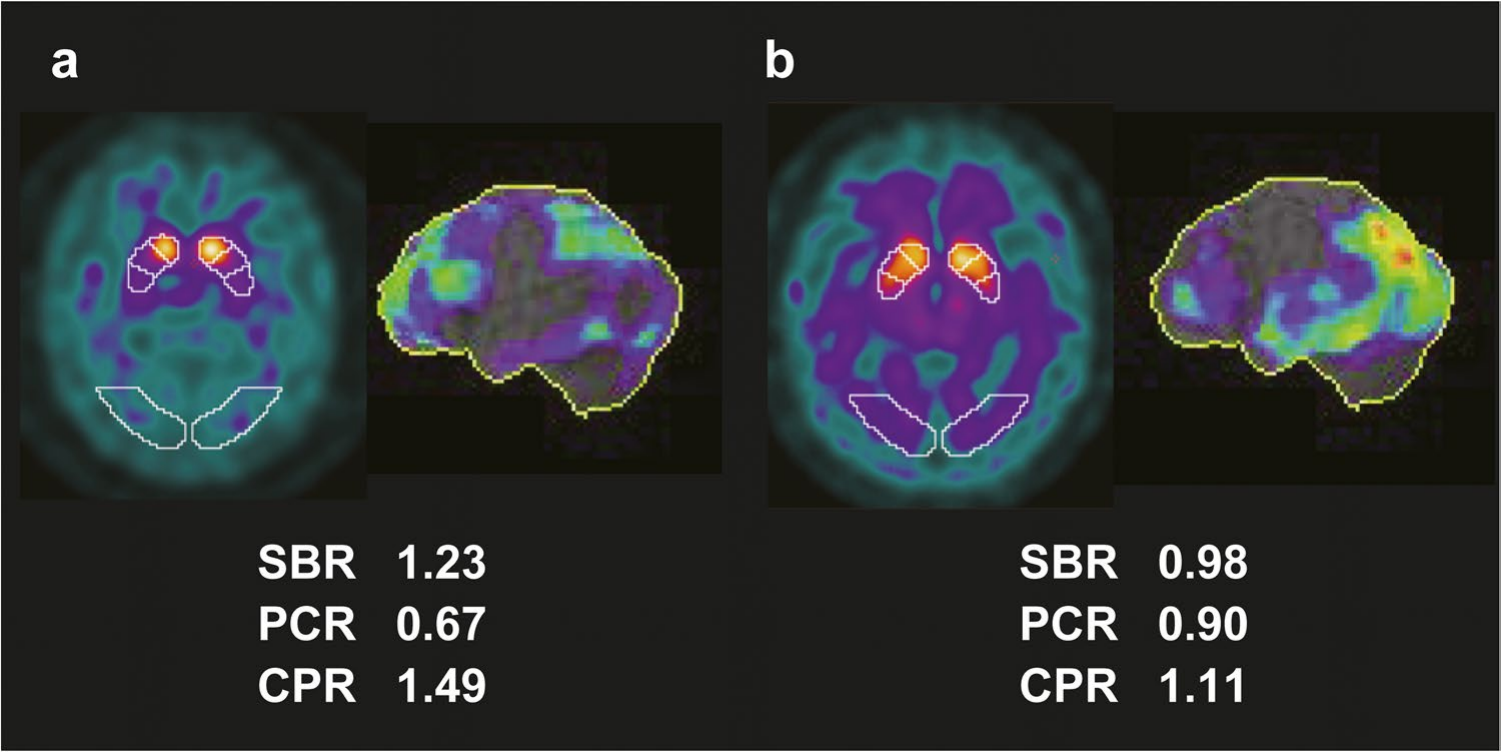
DAT-SPECT and brain perfusion SPECT images of representative cases of PD and DLB. A commercially available software package DaTQUANT was used for VOI-based analysis. The brain perfusion image was visualised with 3D-SSP. **a** A representative case of PD (aged 66 years, female) shows striatal uptake deterioration, especially in the posterior part. **b** A representative case of DLB (aged 76 years, male) shows diffuse striatal uptake deterioration and remarkable hypoperfusion in the temporoparietal lobe. DAT, dopamine transporter; DLB, dementia with Lewy body; PD, Parkinson’s disease; SPECT, single-photon emission computed tomography; VOI, volume of interest; 3D-SSP, three-dimensional stereotactic surface projection

Figure 2 shows representative images of DAT-SPECT and brain perfusion SPECT in patients with PD and DLB, and the calculated quantitative indices are also shown. PD cases tended to have lower PCR values, while DLB cases tended to have lower CPR values.

**Fig. 3:**
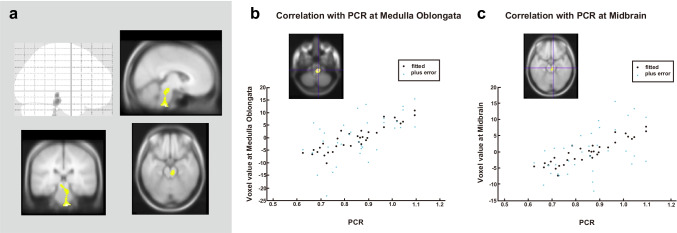
Correlations between brain perfusion SPECT and the PCR index. The PCR index correlated with brain hypoperfusion in the brain stem (medulla oblongata and midbrain) (uncorrected *p* < 0.001, *k* > 100 voxels). Regression lines at notable regions are shown. PCR, putamen-to-caudate ratio; SPECT, single-photon emission computed tomography

**Fig. 4:**
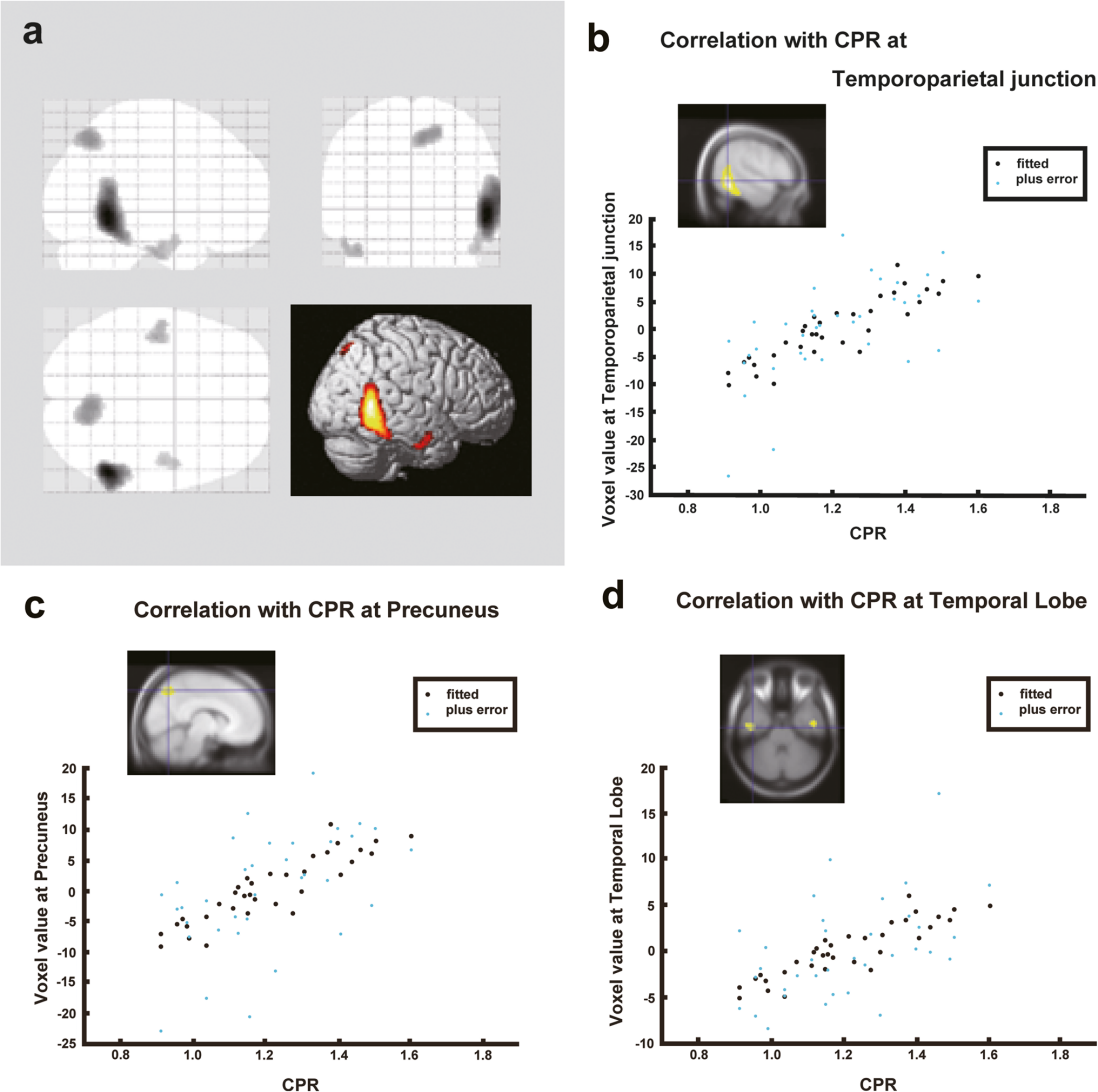
Correlations between brain perfusion SPECT and the CPR index. The CPR index correlated with brain hypoperfusion in the right temporoparietal cortex (FEW corrected *p* < 0.05), right precuneus (uncorrected *p* < 0.001, *k* > 100 voxels), and bilateral temporal cortex (uncorrected *p* < 0.001, *k* > 100 voxels). Regression lines at notable regions are shown. CPR, caudate-to-putamen ratio; SPECT, single-photon emission computed tomography

**Fig. 5:**
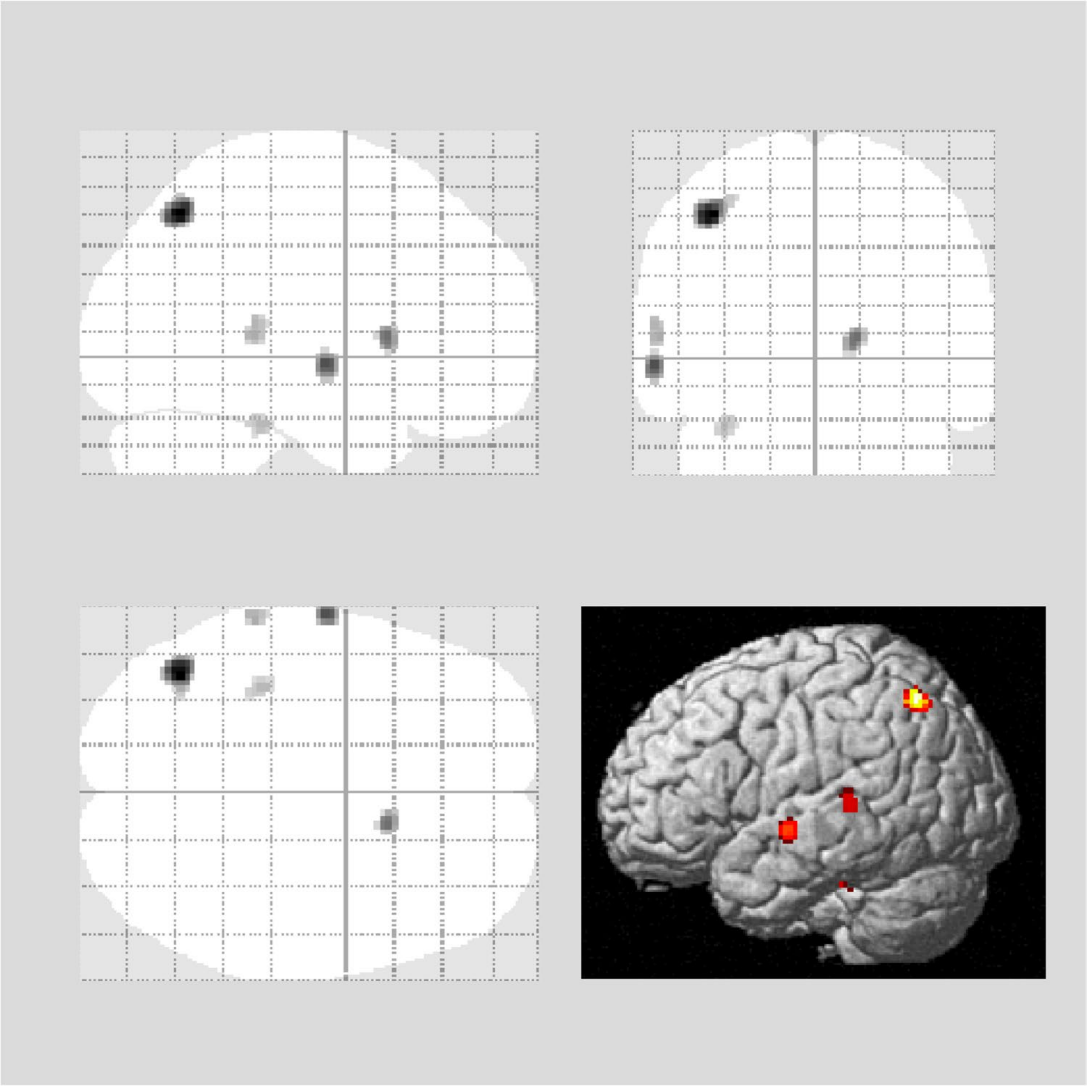
Correlations between brain perfusion SPECT and the MMSE scores. The MMSE scores correlated with brain hypoperfusion in the left temporoparietal cortex (uncorrected *p* < 0.001). Regression lines at notable regions are shown. MMSE, Mini-Mental State Examination; SPECT, single-photon emission computed tomography

Figures 3, 4, and 5 show the results of the SPM analysis. The PCR/CPR indices showed significant correlations with brain perfusion (Figs. 3 and 4), while no significant correlation was observed between the SBR index and brain perfusion (data not shown). Decreased PCR index correlated with hypoperfusion in the brainstem (medulla oblongata and midbrain) (uncorrected *p* < 0.001, *k* > 100; Fig. 3), and decreased CPR index correlated with hypoperfusion in the right temporoparietal cortex (FWE corrected *p* < 0.05), right precuneus (uncorrected *p* < 0.001, *k* > 100), and bilateral temporal cortex (uncorrected *p* < 0.001, *k* > 100; Fig. 4). A slight correlation between the MMSE score and brain perfusion was observed in the left temporoparietal cortex (uncorrected *p* < 0.001; Fig. 5). Table 2 depicts the brain regions showing a significant correlation with the PCR/CPR indices.

**Table 2:** Brain areas showing a significant correlation with the PCR and CPR indices

Area	Cluster size	Coordinates					*Z*-score	*p* values (uncorrected, *k* > 100)
		X		Y		Z		
PCR								
Medulla oblongata	165	8	− 30		− 52		3.88	< 0.001
Mid Bbrain	168	8	− 24		− 18		3.41	< 0.001
CPR								
R temporoparietal lobe	1295	54	− 54		− 4		4.26	< 0.05 (FWE corrected)
R precuneus	427	8	− 66		50		3.52	< 0.001
L temporal lobe	137	− 42	− 16		− 32		3.41	< 0.001
R temporal lobe	106	48	− 10		− 30		3.29	< 0.001

*CPR*, caudate-to-putamen ratio; *FWE*, family-wise error; *L*, left; *MMSE*, mini-mental state examination; *PCR*, putamen-to-caudate ratio; *R*, right

## Discussion

This study showed that decreased putaminal uptake of ^123^I-ioflupane in DAT-SPECT significantly correlated with hypoperfusion in the brainstem; this correlation may reflect movement disorders in patients with LBD. Hacker et al. also demonstrated that midbrain-striatum functional connectivity was reduced in patients with PD, and in their study, striatal functional connectivity with the brainstem was graded as follows: posterior putamen > anterior putamen > caudate [23]. In addition, previous studies have shown more severe substantia nigra cell loss in PDD than in DLB, which consequently leads to more advanced parkinsonism [24]. The Hoehn and Yahr stage or Unified PD rating scale (motor part only) score has been reported to correlate with putaminal uptake of ^123^I-ioflupane in patients with PD [12]. These findings indicate that the midbrain-putamen dopaminergic connectivity contributes to motor dysfunction in patients with LBD. Thus, these findings represent a possibility that decreased PCR index could indicate enhanced severity of movement disorders caused by dysfunction of the putamen dopaminergic system connected from the brainstem.

We also demonstrated that decreased caudate uptake of ^123^I-ioflupane in DAT-SPECT was significantly correlated with hypoperfusion in the right temporoparietal cortex, right precuneus, and bilateral temporal lobes. Consistently, previous studies have shown that glucose hypometabolism in the temporoparietal areas correlates with dementia severity in LBD [25, 26] and that the caudate uptake of ^123^I-ioflupane correlates with cognitive impairment [25–30]. Our results and these previous findings indicate that the correlation between decreased CPR index and hypoperfusion in the temporoparietal cortex may reflect cognitive impairment in patients with LBD. In particular, a remarkable correlation (FWE corrected *p* < 0.05) was observed between decreased CPR index and hypoperfusion in the right temporoparietal region in patients with LBD. Patients with PD and DLB are more likely to have visuospatial dysfunction than those with Alzheimer’s disease [31, 32], and previous studies have reported that visuospatial dysfunction presents as right-dominant hypoperfusion or hypometabolism in the brain [33–35]. In addition, Marquie et al. demonstrated that reduced caudate DAT concentration was associated with worse visuospatial ability in patients with DLB [36]. These findings indicate that significant hypoperfusion in the right temporoparietal region may be indicative of the visuospatial dysfunction observed in patients with LBD. Our results also showed that hypoperfusion in the precuneus is correlated with decreased caudal uptake of ^123^I-ioflupane on DAT-SPECT; however, the posterior cingulate was spared from this hypoperfusion. This finding represents a characteristic sign, known as the “cingulate island sign,” observed on the brain perfusion SPECT of patients with DLB [37]. Early caudate dopaminergic dysfunction is reportedly also a predictor of future cognitive impairment [38–40]. Cognitive decline and related symptoms are not a consequence of α-synuclein-induced neurodegeneration alone because amyloid β and tau pathologies also contribute to overall deficits [41–45]. These pathological changes may synergistically influence clinical features, including a shorter duration or a more malignant course [46, 47]. Therefore, we speculate that the CPR index has the potential to indicate the severity of cognitive impairment and clinical course in patients with LBD.

This study found that the SBR index had no significant correlation with brain perfusion. This result suggests that, unlike the PCR/CPR indices, the SBR index, which represents the magnitude of striatal uptake of ^123^I-ioflupane, has little advantage for assessing the symptoms of motor/cognitive dysfunctions in patients with LBD.

We demonstrated that global cognitive impairment measured using the MMSE correlated with left temporoparietal cortex hypoperfusion in patients with LBD. Moreover, left-sided lateralisation has been observed between brain perfusion and the MMSE score in patients with Alzheimer’s disease [48, 49], probably because most of the items of the MMSE questionnaire rely on left hemispheric cognitive processing.

Some patients included in this study overlapped with those included in our previous studies [10, 15]. In our previous studies, we assessed the combined diagnostic utility of the quantitative indices of DAT-SPECT or iodine-131-meta-iodobenzylguanidine scintigraphy for PD and atypical parkinsonian syndromes; however, we did not use brain perfusion SPECT or assess the correlation between brain perfusion and DAT-SPECT in LBD [10, 15]. Nobili et al. previously evaluated the correlation between brain perfusion and DAT-SPECT in patients with PD and found that brain perfusion in the posterior cingulate, parahippocampal gyrus, and middle temporal gyrus correlated with uptake in the caudate, whereas that in the posterior cingulate, parietal precuneus, and occipital cuneus correlated with uptake in the putamen [50]. However, their study enrolled only patients with PD, and they used the SBR of the caudate/putamen regions, not the PCR or CPR, as quantitative indices. The PCR/CPR indices have been suggested to be particularly valuable as they are background-, age-, and camera-independent [6]. These factors may explain the difference between our findings and theirs.

This study has some limitations. First, the number of patients analysed was relatively small. Additional studies with larger numbers of participants are needed to confirm our observations. Second, the diagnoses of PD, PDD, and DLB were clinical diagnoses and not pathologically confirmed. Finally, this was a single-centre study, and institution-specific factors might limit the generalizability of our results.

## Conclusion

This study found that changes in striatal tracer accumulation on DAT-SPECT correlated with brain hypoperfusion in several specific regions (brainstem, temporoparietal cortex, or precuneus) in patients with LBD.

## Data Availability

All data generated or analysed during this study are included in this published article.

## Abbreviations

CPR: Caudate-to-putamen ratio
DAT: Dopamine transporter
DLB: Dementia with Lewy bodies
FEW: Family-wise error
LB: Lewy body
LBD: Lewy body diseases
MMSE: Mini-mental state examination
PCR: Putamen-to-caudate ratio
PD: Parkinson’s disease
PDD: Parkinson’s disease with dementia
SBR: Specific binding ratio
SPECT: Single-photon emission computed tomography
SPM: Statistical parametric mapping
3D-SSP: Three-dimensional stereotactic surface projection
VOI: Volume of interest
